# BurnCalc assessment study of computer-aided individual three-dimensional burn area calculation

**DOI:** 10.1186/s12967-014-0242-x

**Published:** 2014-09-10

**Authors:** Wen-bo Sheng, Ding Zeng, Yan Wan, Li Yao, Hong-tai Tang, Zhao-fan Xia

**Affiliations:** Department of Burns, Changhai Hospital, Second Military Medical University, Shanghai, China; Department of Burns, The Second Artillery General Hospital, Beijing, China; School of Computer Science and Technology, Donghua University, Shanghai, China

**Keywords:** Three-dimensional scanning, Individualized body model, Burn area estimation

## Abstract

**Background:**

Accurate estimation of a burned area is crucial to decisions about fluid resuscitation, surgical options, nutritional support, and prognosis. Widely used clinical methods to estimate a burn area are two-dimensional. They do not consider age, sex, body mass, physical deformities, or other relevant factors. Computer-aided methods have improved the accuracy of estimating burned areas by including data analysis and reducing subjective differences. Three-dimensional (3D) scanning allows us to determine body dimensions rapidly and reproducibly. We describe an individualized, cost-efficient, portable 3D scanning system, BurnCalc, that can create an individual 3D model and then calculate body surface area (BSA) and the burn area accurately and quickly.

**Methods:**

The BurnCalc system was validated by verifying the accuracy and stability of BSA calculation. We measured 10 regular objects in experiment 1, using Student’s t-test and the intraclass correlation coefficient (ICC) in the analysis. In experiment 2, artificial paper patches of known dimensions were attached to various parts of the body of 40 volunteers. Their sizes were then calculated using BurnCalc. The BurnCalc data were compared to actually measured values to verify accuracy and stability. Total BSAs of these 40 volunteers were also calculated by BurnCalc and compared to those derived from an accepted formula. In experiment 3, four experts using Chinese Rule-of-Nines or Rule-of-Palms methods calculated the percentages of the total BSA in 17 volunteers. Student’s t-test and ICC, respectively, were used to compare the results obtained with the BurnCalc technique.

**Results:**

Statistically, in experiment 1, *p =* 0.834 and ICC = 0.999, demonstrating that there was no difference between the BurnCalc and real measurements. Also, the hypothesis of null difference among measures (experiment 2) was true because *p >* 0.05 and ICC = 0.999, indicating that calculations of the total BSA and the burn area were more accurate using the BurnCalc technology. The reliability of the BurnCalc program was 99.9%. In experiment 3, only the BurnCalc method exhibited values of *p* > 0.05 (*p =* 0.774) and ICC = 0.999.

**Conclusions:**

BurnCalc technology produced stable, accurate readings, suggesting that BurnCalc could be regarded as a new standard clinical method.

## Background

Burn area estimation helps determine fluid resuscitation, nutrition support, surgical decisions, and prognosis [1,2]. The size of the burned area is expressed as a percentage of the total body surface area (%TBSA). For the past eight decades, medical professionals have relied on hand-drawn diagrams and other methods or formulas to determine the % TBSA based on numerous reported comparative clinical practices and research projects. Standard two-dimensional (2D) charts (e.g., Lund and Browder: Rule-of-Nines) helped determine the percentages of the burned surface area. Chinese surgeons established the Chinese “Rule-of -Nines” according to clinical practices and continuous revisions for years. The Rule-of-Palms has been used as an alternative method [3,4].

The chart revised by Lund and Browder in 1944 was based on previous methods and is commonly used nowadays because of its simplicity and practicability [5]. Thus, a burned area calculation has played a key role for six decades. The Rule-of-Nines divides the body surface into areas that each represents 9% of the TBSA [3,4]. The Rule-of-Palms is defined as the projection (apparent) area of the hand, which represents 1% TBSA.

Although these formulas are used widely, their weaknesses are well known and have been discussed previously. Human body shapes show enormous variability that is influenced by age, sex (especially women’s breasts [6]), racial characteristics, physical deformities (e.g., limb defects), and the physiological state. None of the common 2D estimation methods are suitable for accurate calculations of burned areas of different body shapes. These methods could provide only rough approximations of % TBSA compared to the actual burned areas, each of which is unique and individual [7]. Even worse, somebody regions cannot be represented, such as the lateral sides of the body. The Rule-of-Nines overestimates % TBSA, especially in persons with a high body mass index (BMI) [7]. BMI has a great influence on the BSA [8]. Previous studies have found that the actual palm surface area (PSA) is 0.76–0.78%, indicating an overestimation of 10–20% by the Rule-of-Palms technique [9-11]. Such overestimation may cause a series of complications, such as pulmonary edema, cerebral edema, and even abdominal compartment syndrome.

A larger issue is the lack of standards. Burn wounds often display irregular shapes and varied distributions. Even the same wound may be viewed differently by different doctors. Another, psychologically based error is that documenters tend to overestimate burn areas, especially in massively burned patients. In such cases, the patient is deemed to have suffered a severe burn injury, necessitating a burn team to achieve a positive result [12]. All of these factors cause injury misjudgments and varied therapy strategies [13]. Thus, to individualize therapies, the data for burned areas need to be comparable and repeatable. Standard procedures are critical to help less experienced burn personnel and for collecting data from different burn centers.

The methods used to estimate the extent of a burned area, although defective, have been applied for many years without improvement because of technical constraints. Today, the innovations in modern computer technology and current demands for individualized and specialized treatment have been significant factors in developing an accurate and individualized estimation technique. Thus, with the aim of overcoming the drawbacks of existing methods, a series of computer-aided 3D models were developed to calculate the extent of a burned area rapidly and reproducibly. Representative efforts are the BurnCase 3D, EPRI 3D Burn Vision, BAI, and Chang Gung Whole Body Scanner.

For both the BurnCase 3D [14-16] and EPRI 3D Burn Vision [12], % TBSA was determined via default stored models in a library adjusted by data input (e.g., age, sex, height). Operators selected similar models and performed burn representation by outlining burn triangles with a paintbrush. The % TBSA was estimated as the ratio of the areas of the triangles selected (burned areas) to the total area of the triangles constituting the whole body. The models support three-dimensional (3D) rotation, zoom, and other functions, providing good intuitive and 3D vision. The drawbacks of these models, however, are that they did not consider obesity or deformity. In contrast, BAI has more than 80 models taking age, sex, and weight (especially obesity) into account to eliminate the above-mentioned drawbacks. Furthermore, accuracy and feasibility have been confirmed in further clinical trials with the BAI models, and the database supports information-sharing and multi-center exchanges [17].

All of these systems have employed the limited default 3D models with different body features, but they could not provide precise individual information. The Chang Gung Whole Body Scanner (CGWBS) is a 3D body scanning system with six scanners in vertical towers. It was created by Yu et al. to scan the body from head to toe in an attempt to build a 3D model [18]. With this technology, the mean PSA/TBSA ratio was found to be 0.89% (SD 0.09%) in adults. It was used to develop a new TBSA calculation formula via scanning 3951 Asian subjects [SA = 0.015925(Ht*Wt)^1/2^] [19]. The drawbacks for clinical application of CGWBS were the complexity of its operating system, high cost, and unwieldiness.

Based on these efforts, an accurate, cost-efficient, portable 3D body scanning system, known as BurnCalc, was developed. Individualized 3D models could be built using this model, and burned areas and TBSA can be calculated accurately and quickly.

## Methodology

### Measurement protocol

**Figure 1:**
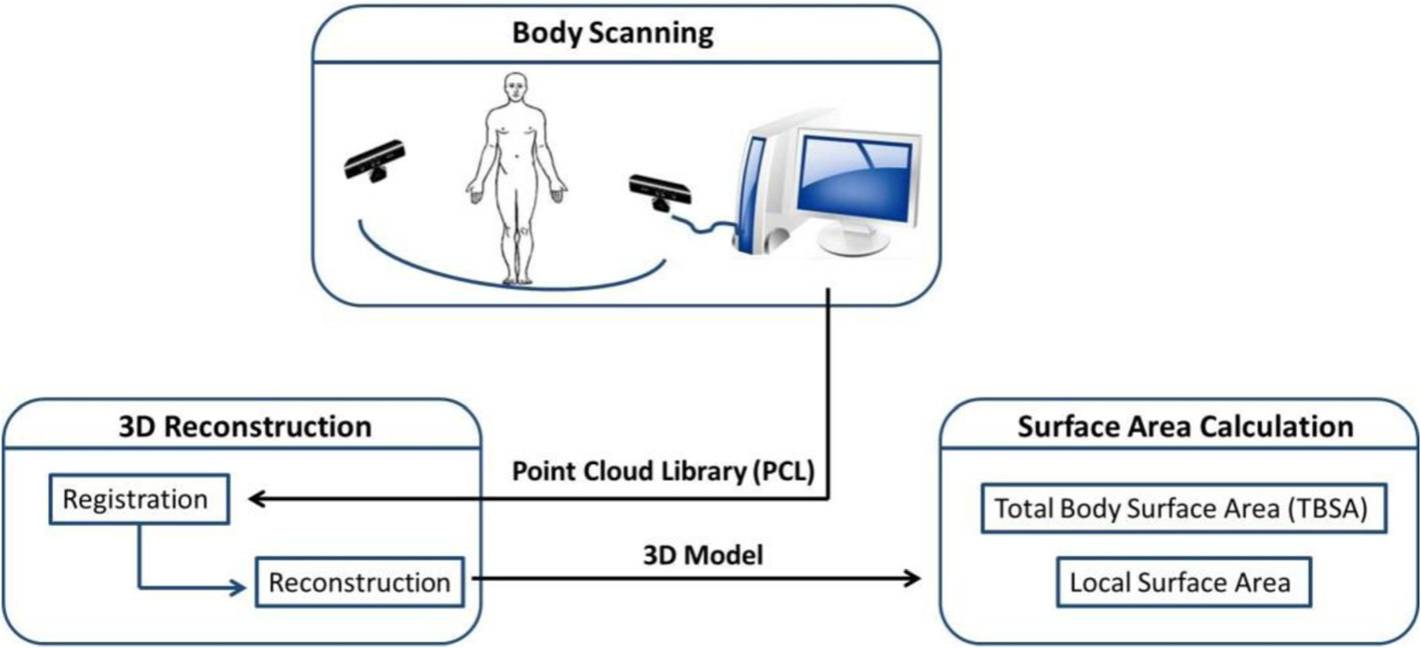
**Framework for BurnCalc based on Kinect 3D scanning. **The system consists of three subsystems: 3D body scanning, 3D reconstruction and interactive surface area calculation.

The BurnCalc system is divided into three subsystems: 3D scanning; 3D reconstruction; interactive surface area calculation. The framework of BurnCalc, based on Kinect® 3D scanning, is presented in Figure 1. BurnCalc was conducted in a personal computer. The CPU is Intel(R) CORE i7-2600, and the VGA card is NVIDIA GTX660 with 2G video memory. Ethical approval was given by the Shanghai Changhai Hospital Ethics Committee with the following reference number: CHEC2013-140.

### Three-dimensional scanning

The subsystem for 3D scanning is aimed at obtaining 3D images of an object’s surfaces. The hardware of the 3D scanning system consists of an imaging system, a 3D data collection system, and a calibration system. The scanning system is Kinect®, which is a hand-held scanner consisting of three lenses. Two of the lenses are used to obtain depth information (spatial coordinate) to code the object. One is an infrared transmitter, sending infrared structured light. The other is an infrared CMOS camera that responds to different intensities of light. The depth camera uses a light coding method to encode the target space [20]. The infrared transmitter emits a matrix according to certain rules. The CMOS sensor captures the matrix, which changes with the spatial depth. The third lens is an RGB camera (resolution 640 × 480) to collect color information simultaneously. Kinect fulfills the primary goal of acquiring a model as completely as possible in one pass, allowing slight movement. Holding the Kinect scanner, the operator walks around an object slowly - at a distance of 50 cm - to collect its image information. The total time for scanning is about 2 minutes. The body surface of each volunteer was measured in any posture. Both male and female volunteers were required to wear shorts. In addition, the females wore a sports bra during the scanning process.

### Three-dimensional reconstruction

The points in every frame, including their depth and color information, were combined as depth data. Depth data of all frames were stored in a Point Cloud Library (PCL) of a software platform for recall and editing. The PCL is a large-scale, open project for 2D/3D imaging and point cloud processing. The 3D reconstruction was achieved by gridding 3D coordinates and RGB information. The depth data in the PCL were independent and isolated, and contained unwanted background information and noise data as well. It was difficult to handle the sampling data directly because it consisted of thousands of discrete points in a 3D coordinate. Hence, a continuous, accurate, smooth 3D surface model was necessary.

**Figure 2:**
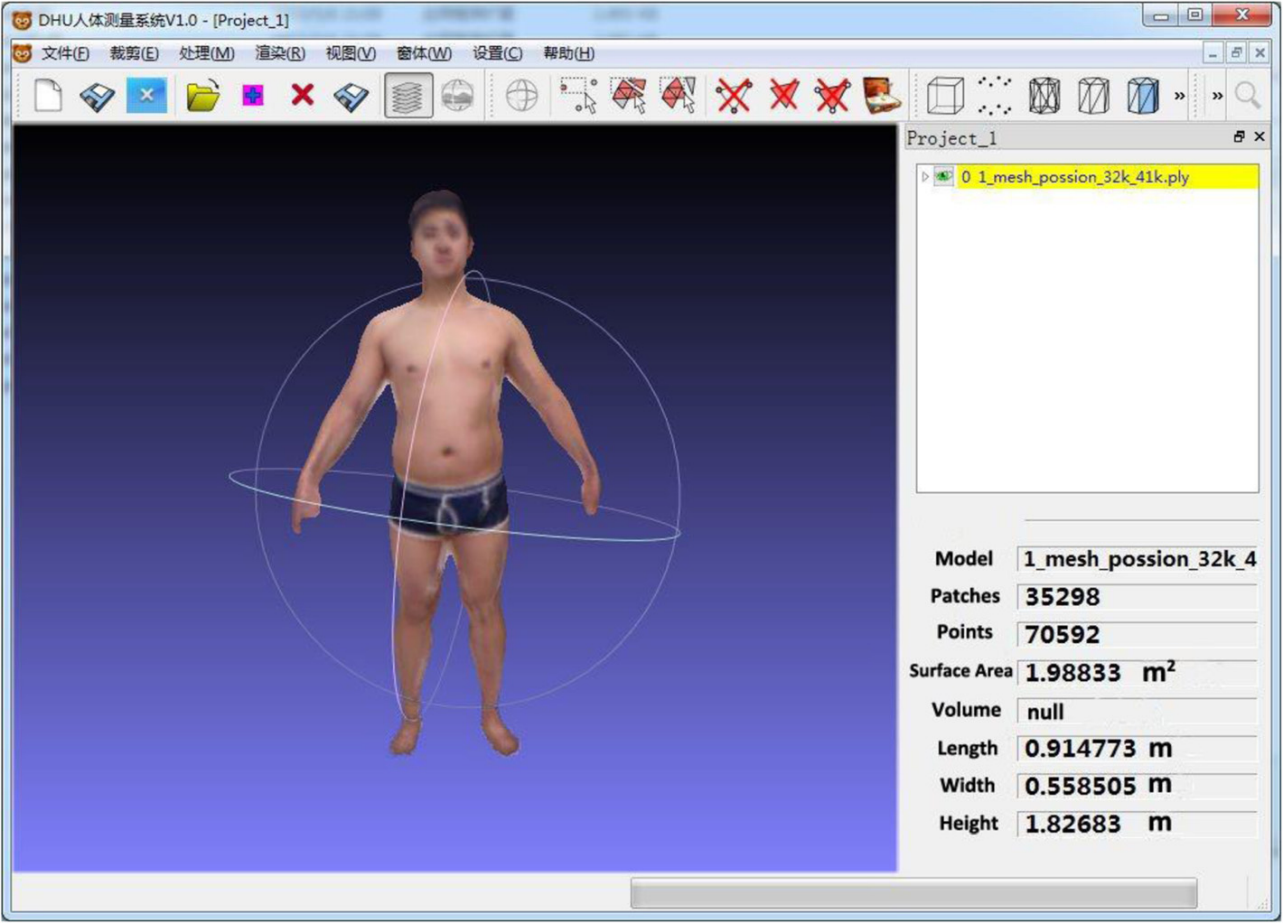
**Example of the interface of BurnCalc.** The model was obtained by wiping off the background and eliminating interference in a visualization platform.The individual 3D model can be translated, rotated and scaled along the 3 axes. The results of calculation was presented on the right.

Early on, Besl et al. [21] proposed an approach of points registration based on contour features called the iterative closest point (ICP) algorithm. It was used to handle registration of 3D models accurately and efficiently. However, a desired geometry was needed before the observation model was built. With the emergence of the Kinect device, Izadi et al. [22] proposed KinectFusion techniques, which improved the traditional ICP algorithm and used volumetric integration to build 3D scene information. This adjustment made it easy to reconstruct a sophisticated 3D scene quickly just by moving the Kinect device. Because of movement during the scanning, the depth data were different, and each frame could have repeated data from the previous frame. Hence, the current frame data had to be matched with the previous frame’s data to piece it all together, a process called registration. With this 3D reconstruction method, then, real-time data from a Kinect sensor were handled in real time. The proposed reconstruction of the entire model was based on KinFu from PCL open-source projects. A smooth, sophisticated 3D body model could be quickly reconstructed using the PCL and its KinFu, which improved the ICP and KinectFusion algorithm. An individual 3D model was obtained by eliminating the background and thus the interference in a visualization platform on which the model could be translated, rotated, and scaled along the three axes (Figure 2). The amount of time needed to form the smooth 3D model depended on the number of data. This step usually took 3–5 minutes. The whole process would then take about 10–15 minutes including further model editing.

### Interactive surface area calculation

The visualization interactive soft platform supplies the function of a designated surface area calculation. The BSA is computed by accumulating the triangles on the model, which are composed of three adjacent vertices. The area of each triangle is calculated using Heron’s formula.

The area of a single triangular mesh patch, *Fi*, is calculated by Heron’s formula with each side length through their vertex coordinates.

$$ {\mathrm{S}}_{{\mathrm{F}}_{\mathrm{i}}}=\sqrt{P_{F_i-p1p2p3}\left({P}_{F_i-p1p2p3}-{D}_{F_i-p1p2}\right)\left({P}_{F_i-p1p2p3}-{D}_{F_i-p1p3}\right)\left({\mathrm{P}}_{F_i-\mathrm{p}1p2p3}-{D}_{F_i-p2p3}\right)} $$

The whole surface area is $\mathrm{S}={\displaystyle {\sum}_1^{\mathrm{n}}}{{\ \mathrm{S}}_{\mathrm{F}}}_{{}_{\mathrm{i}}}$.

${F_i}_{\hbox{-} p1}\left({x}_{Fi-p1},{y}_{F_{i\hbox{-} p1}},\ {z}_{F_{i\hbox{-} p1}}\right),\ {F}_{i\hbox{-} p2}\left({x_F}_{{{}_{i\hbox{-} p}}_2},{y}_{F_{i\hbox{-} p2}},{z}_{F_{i\hbox{-} p2}}\right)$ and ${F}_{i\hbox{-} p3}\left({x_F}_{{}_{i\hbox{-} p3}},{y}_{F_{i\hbox{-} p3}},{z}_{F_{i-p3}}\right)$ are the three coordinates of the triangular mesh patch Fi. Also, p1, p2, and p3 are the three vertices of Fi. ${D}_{F_{i\hbox{-} p1p2}},{D}_{F_{i-p1p3}},{D}_{F_{i\hbox{-} p2p3}}$ represents each side length, $P=\left({D}_{F_{i\hbox{-} p1p2}}+{D}_{F_{i\hbox{-} p1p3}}+{D}_{F_{i\hbox{-} p2p3}}\right)/2$.

To obtain the burn contour and compute the area, it is necessary to extract features of the burned area. The edge of the featured area is outlined on the 3D model by choosing the points and drawing a line between adjacent points. The lines between the chosen points form a closed area. The area is then computed as described above. The system finds all of the triangles inside the featured area, eliminates the reduplicative triangles, and calculates the areas of the remaining triangles.

**Figure 3:**
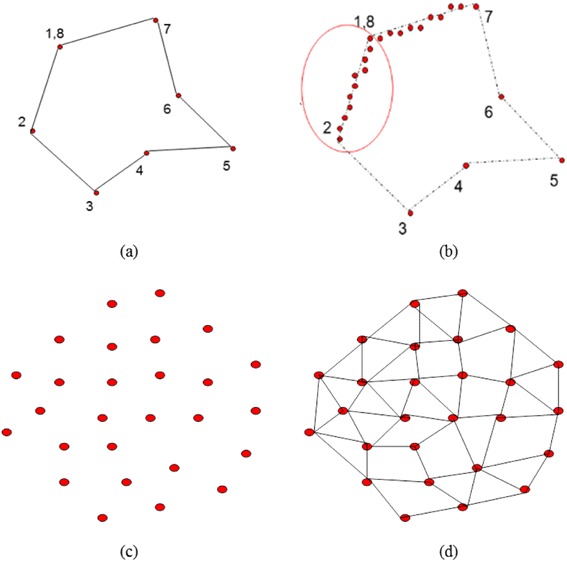
**Procedure of marking the feature region. a**. Outline the edge of the featured area. **b**. Identify all the points near the lines. **c**. Identify all of the interior points of the featured area in the smallest rectangular region. **d**. Compute the size of the featured area.

The procedure for marking the featured region is as follows (Figure 3).

- Outline the edge of the featured area manually by choosing the points and drawing a line between each two adjacent points. If the first point is not the last point or a closed area is not available, the area must be formed manually.
- Identify all the points near the lines drawn previously as the points on the edge of the featured area.
- Identify all of the interior points of the featured area in the smallest rectangular region.
- Compute the size of the featured area. Identify all the triangles of the featured area, eliminate the reduplicative triangles, and calculate the areas of the remaining triangles. It should be noted that when the area is calculated it is necessary to remove all of the edge points of the featured region; otherwise, the surface area is extended.

### Test approaches and experimental design

#### Experiment 1: Evaluate the accuracy and stability of BurnCalc for calculating the surface area

**Figure 4:**
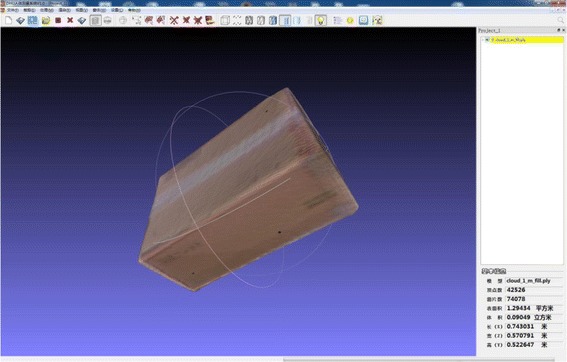
**The cube used in Section 2.2.1 for evaluation the accuracy and stability of BurnCalc.**

The aim of experiment 1 was to identify the measurement error of 3D scanning and the computational error of the software platform. In this experiment, 10 cubes of different sizes were scanned, calculated, and their exact surface areas measured by both BurnCalc and manually (Figure 4). The correlation between BurnCalc calculations and the real (manual) measurements was achieved by Student’s t-test.

#### Experiment 2: Compare burned areas and TBSA calculated by BurnCalc with the gold standard

**Figure 5:**
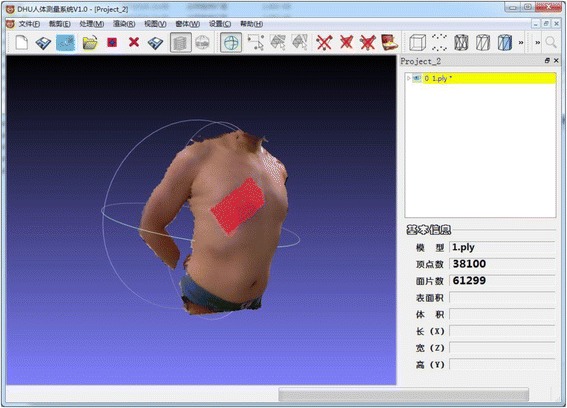
**Example of the patch used as simulated burn area.**

For experiment 2, the area of the burned area according to BurnCalc was compared with that determined with the gold standard measurement. In all, 40 subjects of different heights, ages, and weights were selected. Simulated burned area patches (measured areas with different shapes and colors) were attached to different body parts and estimated with BurnCalc (Figure 5). The intraclass correlation coefficient (ICC) was calculated, and Student’s t-test was applied to verify correlations between BurnCalc calculations and real measurements. Also, the TBSA data obtained during the scanning process were compared with the data obtained using the 3D-derived formula of Yu et al. (described earlier).

#### Experiment 3: Compare % TBSA calculated by traditional methods with data derived from the BurnCalc system and the gold standard

The reliability of traditional methods—Rule-of-Nines or Rule-of-Palms—was compared with that of the gold standard and BurnCalc. The same patches were used. A total of 17 differently sized patches were measured by four burn surgeons from the Department of Burns of Changhai Hospital, estimating the % TBSA of the patches by the Chinese Rule-of-Nines or the Rule-of-Palms. The % TBSA of those patches were also calculated with BurnCalc. The real value of the % TBSA was defined as the patch area divided by 0.015925(Ht*Wt)^1/2^. The BurnCalc value was the ratio of the calculated areas of patches to the calculated BSA. The ICC represented the reliability percentage if only one method was used to measure the body surface. Student’s t-test was applied as well.

#### Statistical analysis

Data were all tested by normal distribution. We performed Student’s t-test for the statistical analysis of the real calculated values compared to the scanned values or measured values. The statistical significance was considered at *p <* 0.05. The ICC was also calculated to represent the reliability percentage if the scanning method or traditional methods were used to measure the body surface. The mean absolute error (MAE) was calculated to measure how far the estimates of the BurnCalc were from the real values. The statistical package used was SPSS Version 17.0 (SPSS, Chicago, IL, USA).

## Results

### Experiment 1: accuracy of BSA calculation

**Figure 6:**
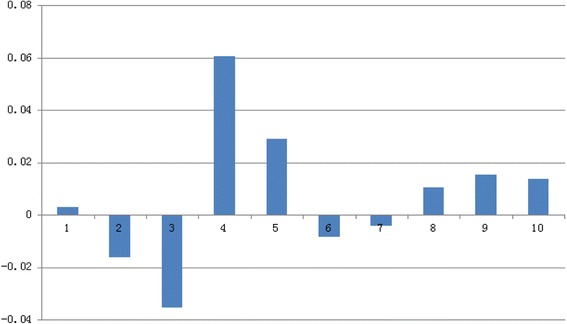
**Difference in % between real values and BurnCalc of cubes in Table** **1** **.**

The comparison of BurnCalc calculations with real values is shown in Table 1. These data were analyzed with Student’s t-test. We hypothesized that there was no significant difference between the real values and the measurements with BurnCalc. As t = 0.216 and *p* = 0.834 (>0.05), the initial hypothesis can be considered as true. MAE was found to be 107.96. The differences between BurnCalc and real values are shown as percentages in Figure 6.

**Table 1:** **Comparison between real measures and calculated area with BurnCalc of 10 different cubes**

**n**	Real values*	**BurnCalc calculated area**	**Difference**
**1**	8833.87	8807.36	26.52
**2**	7264.23	7382.79	−118.56
**3**	4252.64	4402.80	−150.16
**4**	2866.30	2692.47	173.83
**5**	4693.28	4557.56	135.72
**6**	33740.49	34027.50	−287.01
**7**	7582.63	7614.47	−31.84
**8**	5954.83	5892.51	62.32
**9**	3739.20	3681.93	57.27
**10**	2624.74	2588.39	36.35
**Mean (μ)**	8155.22	8164.78	−9.56
**Standard deviation (σ)**	9227.41	9331.39	140.20

*Cube surface area = L*H + L*W + H*W (L: length, H: height, W: width).

Measures are presented in cm^2^.

### Experiment 2: comparison of burned areas and BSA: BurnCalc versus the gold standard

**Figure 7:**
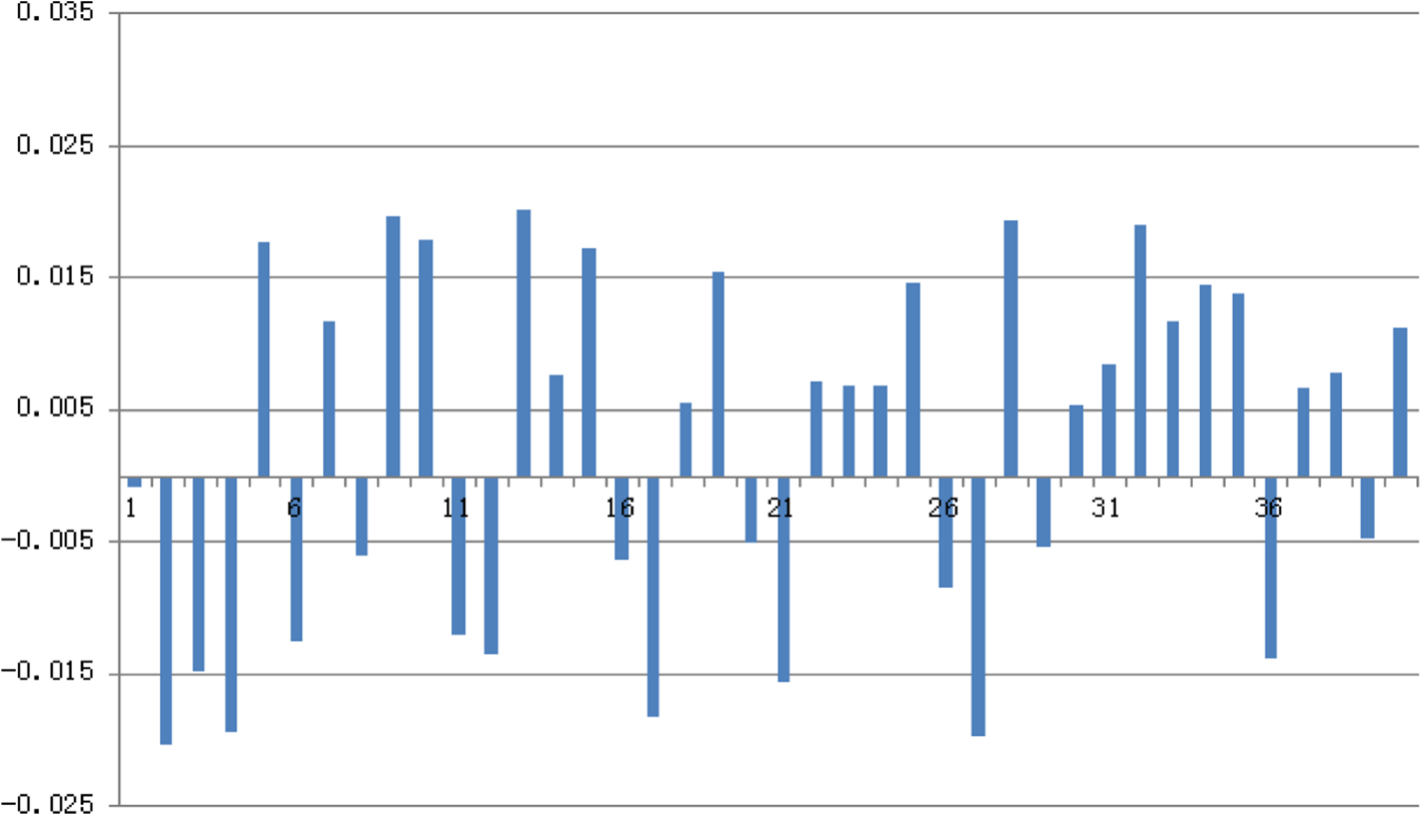
**Difference in % between real values and BurnCalc of 40 patches in Table** **2** **.**

**Figure 8:**
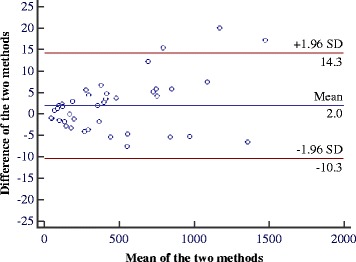
**Bland-Altman diagram for data summarized in Table** **2** **.**

The comparison of real values of 40 patches and the TBSA as calculated by BurnCalc are shown in Tables 2 and 3, respectively. As *t* = −1.975 and *p* = 0. 055 (>0.05), there was no significant difference between the real values and those found with BurnCalc. For the data in Table 2, the ICC = 0.999, and MAE = 4.86. For the data in Table 3, *t =* −0.673, *p* = 0.505 (>0.05), ICC = 0.990, and MAE =168.96. These values corroborated the fact that BurnCalc calculations were correlated with the real values. The differences between BurnCalc and real values of patches and total body surfaces are presented as percentages in Figures 7 and 8, respectively.

**Table 2:** **Comparison between real measures and BurnCalc of the 40 patches**

**n**	Real values*	**BurnCalc calculated area**	**Difference**
1	170.00	169.85	−0.15
2	52.00	50.94	−1.06
3	101.25	99.75	−1.50
4	148.50	145.62	−2.88
5	375.85	382.47	6.62
6	297.40	293.65	−3.75
7	69.50	70.31	0.81
8	200.00	198.79	−1.21
9	785.45	800.80	15.35
10	685.50	697.72	12.22
11	445.20	439.83	−5.37
12	558.85	551.27	−7.58
13	275.80	281.33	5.53
14	478.00	481.62	3.62
15	1160.50	1180.52	20.02
16	844.60	839.21	−5.39
17	182.70	179.37	−3.33
18	750.50	754.64	4.14
19	188.50	191.40	2.90
20	367.80	365.93	−1.87
21	269.60	265.38	−4.22
22	726.40	731.50	5.10
23	847.50	853.30	5.80
24	1084.00	1091.37	7.37
25	294.20	298.50	4.30
26	558.50	553.71	−4.79
27	48.50	47.54	−0.96
28	98.50	100.40	1.90
29	974.65	969.32	−5.33
30	357.50	359.40	1.90
31	408.30	411.72	3.42
32	118.35	120.60	2.25
33	1465.25	1482.40	17.15
34	86.85	88.10	1.25
35	123.60	125.30	1.70
36	136.65	134.74	−1.91
37	397.50	400.10	2.60
38	744.55	750.30	5.75
39	1360.50	1353.88	−6.62
40	416.50	421.15	4.65
**Mean (μ)**	466.38	468.34	1.96
**Standard deviation (σ)**	374.00	376.57	6.28

*Patches area were manually measured and calculated.

Measures are presented in cm^2^.

**Table 3:** **Comparison between calculated BSA with formula and BurnCalc of 40 human bodies**

**n**	Formula calculated area*	**BurnCalc calculated area**	**Difference**
**1**	18617.27	18864.49	247.22
**2**	19268.59	19517.22	248.63
**3**	19852.03	19573.70	−278.33
**4**	17886.41	18020.64	134.23
**5**	18225.60	18003.77	−221.83
**6**	18554.50	18313.06	−241.44
**7**	15398.74	15524.40	125.66
**8**	19288.32	19094.17	−194.15
**9**	17114.75	17354.91	240.16
**10**	19931.08	20014.38	83.30
**11**	19465.01	19213.47	−251.54
**12**	19056.84	18974.09	−82.75
**13**	15170.60	15413.48	242.88
**14**	17260.82	17018.84	−241.98
**15**	18734.73	18606.92	−127.81
**16**	19311.32	19470.82	159.50
**17**	15988.57	16014.38	25.81
**18**	19131.22	18998.47	−132.75
**19**	20437.43	20193.53	−243.90
**20**	16635.34	16881.33	245.99
**21**	18220.04	18319.35	99.31
**22**	17625.75	17703.93	78.18
**23**	16868.48	17010.78	142.30
**24**	16224.75	16031.44	−193.31
**25**	16349.32	16507.81	158.49
**26**	16895.52	17021.33	125.81
**27**	16708.35	16912.57	204.22
**28**	16529.05	16397.04	−132.01
**29**	18244.38	18017.53	−226.85
**30**	16936.00	17014.27	78.27
**31**	18824.54	18697.59	−126.95
**32**	17045.70	17243.71	198.01
**33**	18711.71	18552.95	−158.76
**34**	18704.25	18574.94	−129.31
**35**	17566.65	17913.30	346.65
**36**	18192.18	18217.83	25.65
**37**	15893.12	16014.66	121.54
**38**	17225.52	17462.83	237.31
**39**	17260.82	17426.05	165.23
**40**	18967.47	19007.74	40.27
**Mean (μ)**	17858.07	17877.84	19.77
**Standard deviation (σ)**	1320.99	1247.69	185.74

*The formula for body surface area (BSA): SA = 0.015925(Ht*Wt)^1/2^.

Measures are presented in cm^2^.

**Figure 9:**
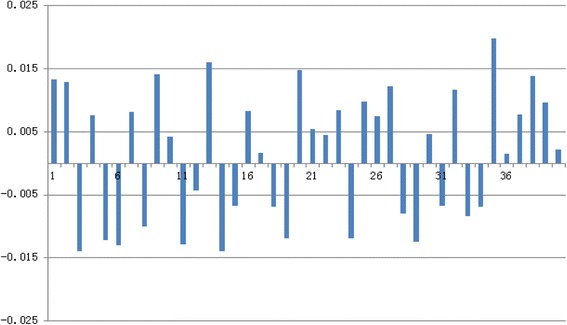
**Difference in % between real values and BurnCalc of 40 human bodies in Table** **3** **.**

**Figure 10:**
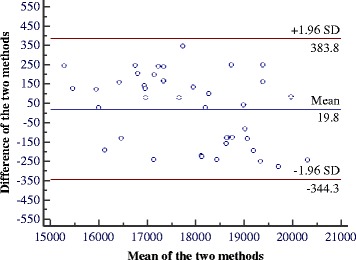
**Bland-Altman diagram for data summarized in Table** **3** **.**

When a Bland and Altman diagram representing the difference between the area measured with BurnCalc and the real values versus the average of both quantities are assessed graphically, a similarity of the two measurements are apparent [23]. Figures 9 and 10 represent the data summarized in Tables 2 and 3, respectively. Horizontal lines represented the 95% confidence intervals according to Student’s t-test. Presuming that the difference between the two measures has a normal distribution, the confidence interval represents the range of values in which the real mean difference would fall with 95% probability.

### Experiment 3: comparison of % TBSA calculated by traditional methods versus BurnCalc and the gold standard

We demonstrated that BurnCalc achieved accurate calculations of the burned area and TBSA in experiments 1 and 2. Differences of % TBSA among areas calculated with BurnCalc and with the Chinese Rule-of-Nines and Rule-of-Palms by different doctors are summarized in Table 4. Results of Student’s t-test and the ICC are shown in Table 5. In this experiment, the ICC represented the reliability if only one method was used to measure the % TBSA. It was concluded that there was a significant difference between the BurnCalc and traditional methods. After analyzing the ICC of BurnCalc (ICC = 0.999) in Table 5 and that in Table 1 (ICC = 0.999), we concluded that values of burned areas measured with BurnCalc can be considered almost equal to that of real values, with a 99.9% reliability. Therefore, the advantage of using the BurnCalc method for burned surface estimation was confirmed.

**Table 4:** **Comparison among real calculated TBSA, BurnCalc and estimated TBSA with traditional methods by different doctors**

	Real calculated TBSA*	**BurnCalc**	**Surgeon 1**	**Surgeon 2**	**Surgeon 3**	**Surgeon 4**
1	32.15	31.51	40.00	37.00	38.00	40.00
2	16.33	16.08	21.00	18.00	20.00	22.00
3	5.63	5.73	8.00	8.00	10.00	7.00
4	3.81	3.94	6.00	5.00	6.00	3.50
5	14.21	13.88	17.00	15.00	18.00	17.00
6	8.95	8.71	10.00	10.00	12.00	11.00
7	1.68	1.70	5.00	3.00	4.00	3.00
8	11.81	11.97	12.00	12.00	13.00	12.00
9	2.40	2.44	5.00	3.00	3.00	4.00
10	6.35	6.42	8.00	7.50	7.00	10.00
11	4.50	4.51	6.00	5.00	6.00	7.00
12	19.17	19.12	21.00	19.00	23.00	24.00
13	9.23	9.57	12.00	10.00	11.00	14.00
14	11.48	11.41	14.00	12.00	13.00	16.00
15	9.02	9.03	12.00	12.00	13.00	14.00
16	24.74	25.10	31.00	27.00	26.00	30.00
17	3.88	3.89	5.00	5.00	4.50	6.00
**Mean (μ)**	10.90	10.88	13.71	12.26	13.38	14.15
**Standard deviation (σ)**	8.33	8.23	9.82	9.00	9.24	10.10

* Real calculated TBSA: measured patch area/formula caculated body surface area.

Measures are presented in percentage (%).

**Table 5:** **ICC and t-Student test for data summarized in Table**

	**BurnCalc**	**Surgeon 1**	**Surgeon 2**	**Surgeon 3**	**Surgeon 4**
**ICC**	0.999	0.978	0.990	0.984	0.972
**t-Student**	0.292	−6.041	−4.650	−6.598	−6.134
**p-Value**	0.774	<0.000	<0.000	<0.000	<0.000

## Discussion

Traditional burned area and BSA estimation methods have been applied in medical fields for years, suggesting that they form the basis for decisions regarding fluid resuscitation, skin transplantation, nutritional support, drug dosage, chemotherapy, and hemodialysis [24,25]. Advantages of the traditional methods cannot be ignored, although their drawbacks are obvious. They lack intuitive vision, and the graphic representations are usually two-dimensional, resulting in omission of hidden body regions or those beyond expression, such as the temporoparietal area, axilla, and body sides. Nowadays, patient information is often subjective, limited, and incomplete, represented by two-dimensional figures or words. Wound situations in reference to burns and the processes of change are difficult to reproduce. These drawbacks are associated with difficult retrospective analyses and comparative assessments of the quality of the medical care applied. They also influence summaries of clinical experiences, and do not contribute to improved health care. Therefore, estimation of a burned area is a critical issue, and the development of a representation system for universal use is sorely needed. If one were available, errors due to incorrect assessments or measuring methods would be eliminated.

The advent of 3D anthropometric technology represents a broad development and great progress. The 3D model is used to represent the body and determine the extent of a burned area more precisely, thereby improving its treatment. Three-dimensional models such as EPRI’s 3D and BurnCase 3D employ such variables as age, sex, weight, and height in their calculations. Their main drawback is the limited, predefined body models they use, rather than being able to adapt to varying body shapes and surfaces. Even though BAI has taken BMI and obesity into account, which is closer to reality, it is still not individualized. BMI is just a crude measurement of body fat, and the relation between itself and fatness varies with the muscle composition, age, sex, and ethnicity. Fat distribution is also an important factor. 3D scanning is a new technique for indirectly estimating the TBSA without inconvenience or harm to the patient during the calculation [26]. It determines body dimensions rapidly and reproducibly. 3D scanning is an indirect technique for estimating BSA; whereas determination of BSA with other methods (e.g., “coating”) [27] is time-consuming and/or stressful, 3D scanning is convenient, results are obtained quickly, and the scan is reproducible within the same subject. Yu et al. used 3D scanning technology to redefine a formula for TBSA calculation in Asian subjects, for which the authors obtaining international recognition and verification [28].

To the best of our knowledge, this is the first color 3D scanning system for creating an individual human body model and estimating the extent of a burned area. The only hardware required is Kinect and a laptop to set up the system, with minimal cost. Compared to previous TBSA estimation models, the potential of BurnCalc is that 3D scanning creates a model closely resembling the human body shape and surface. This ability improves the quality and accuracy of BSA calculation significantly—regardless of whether it is for TBSA or localized segmental SA measurements—avoiding large variations among surgeons. BurnCalc also provides an intuitive 3D graphics user interface, allowing models to be scaled, rotated, and stored as electronic information for further study and sharing.

Two sources of error could influence the accuracy of the computation. One is a computational error in 3D reconstruction. The other is a measurement error. When scanning, we assume that the object does not move during the process—without translation or rotation. It is difficult, however, to keep the body still, which affects the measurement. Kinect is a somatosensory scanner that allows slight movement, and movement errors were corrected automatically during pretests. During the measurement, the 3D model is sensitive to the measurement data, and the real values may be inaccurate because of operational errors, which can cause large measurement errors. Another point to be considered is that errors may occur during the manual editing and tracing processes. In this study, only one surgeon processed the data. We hope to analyze the variability among users in the near future.

In clinical practice, some patients cannot remain standing for a long period or lie quietly in bed. For future work, we propose that patients’ bodies be examined part-by-part, the analyses of which will form different model parts. A complete model can then be built by splicing the various parts into one. Currently, the resolution of BurnCalc is not satisfactory. Also, although Kinect can achieve a relatively clear 3D model, it cannot provide face or wound information. We plan to focus on updating the hardware. We are cooperating with a company to manufacture a scanner with higher resolution (2 million pixels) that could provide the sharpness we need. The better resolution could provide a diagnosis of burn depth by dividing the wound into different colors and calculating each area/proportion through manually correction. Thus, the severity of the wound would be represented by different colors on a software platform. Higher resolution could also help calculate the healing rate and the volume of the scar. Further study on the accuracy of the software in the clinical field is necessary.

## Conclusions

BurnCalc is a noninvasive, precise, individual 3D body scanning system that is easy to operate. It has overcome difficulties in previous scanning equipment, making large-quantity measurements possible and greatly reducing the workload. The stable, accurate verification results suggest that BurnCalc could be regarded as a standard clinical method. Our promising results suggest that the improved process of estimating surface areas can influence future burn treatment. Most importantly, the clinical significance of BurnCalc is that it could become a universal technique for evaluating the extent of a patient’s burned area, thereby allowing more objectively based decisions to be made about treatment.

### Consent

Written informed consent was obtained from the patient for the publication of this report and any accompanying images.
